# Segregation, integration and balance in resting‐state brain functional networks associated with bipolar disorder symptoms

**DOI:** 10.1002/hbm.26087

**Published:** 2022-09-26

**Authors:** Zhao Chang, Xinrui Wang, Ying Wu, Pan Lin, Rong Wang

**Affiliations:** ^1^ College of Science Xi'an University of Science and Technology Xi'an China; ^2^ State Key Laboratory for Strength and Vibration of Mechanical Structures School of Aerospace Engineering, Xi'an Jiaotong University Xi'an China; ^3^ National Demonstration Center for Experimental Mechanics Education Xi'an Jiaotong University Xi'an China; ^4^ Center for Mind & Brain Sciences and Cognition and Human Behavior Key Laboratory of Hunan Province Hunan Normal University Changsha Hunan China

**Keywords:** bipolar disorder, fMRI, functional balance, functional connectivity, nested‐spectral partition, calibrated

## Abstract

Bipolar disorder (BD) is a serious mental disorder involving widespread abnormal interactions between brain regions, and it is believed to be associated with imbalanced functions in the brain. However, how this brain imbalance underlies distinct BD symptoms remains poorly understood. Here, we used a nested‐spectral partition (NSP) method to study the segregation, integration, and balance in resting‐state brain functional networks in BD patients and healthy controls (HCs). We first confirmed that there was a high deviation in the brain functional network toward more segregation in BD patients than in HCs and that the limbic system had the largest alteration. Second, we demonstrated a network balance of segregation and integration that corresponded to lower anxiety in BD patients but was not related to other symptoms. Subsequently, based on a machine‐learning approach, we identified different system‐level mechanisms underlying distinct BD symptoms and found that the features related to the brain network balance could predict BD symptoms better than graph theory analyses. Finally, we studied attention‐deficit/hyperactivity disorder (ADHD) symptoms in BD patients and identified specific patterns that distinctly predicted ADHD and BD scores, as well as their shared common domains. Our findings supported an association of brain imbalance with anxiety symptom in BD patients and provided a potential network signature for diagnosing BD. These results contribute to further understanding the neuropathology of BD and to screening ADHD in BD patients.

## Graphical abstract

We applied a hierarchical module analysis to confirm that bipolar disorder (BD) is related to segregation‐integration imbalance in resting brain functional networks and this imbalance underlies the severe anxiety symptoms. The imbalance‐based network measure can generally better predict diverse BD symptoms than classical graph theory analysis, and it is a promising signature for predicting BD severity and screening attention‐deficit/hyperactivity disorder (ADHD) in BD patients.

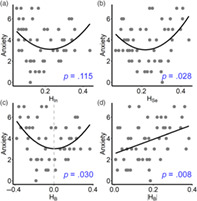

## 1 INTRODUCTION

Bipolar disorder (BD) is a chronic and debilitating mental disorder with a worldwide prevalence of 1%–2% that results in cognitive impairment, a high suicide rate, and a heavy social burden (Association, 2013). BD has a broad range of symptoms including shifts in emotion, mood, and energy; in addition, it mainly involves recurring patterns of (hypo) mania and depression, as well as mixed episodes and variable periods of euthymia (Phillips & Kupfer, 2013). Substantial evidence indicates that BD and other mental disorders, for example, schizophrenia (SZ) and unipolar depression (UD), share the same symptom presentations, risk genes, and cognitive abnormalities (Bora, 2015; Gershon et al., 2011; Yan et al., 2021; Yu et al., 2010). These overlapped symptoms result in great challenges in accurately diagnosing BD in a timely manner (Hirschfeld, 2014; Sendi et al., 2021; Tondo et al., 2017). For example, BD patients spend the majority of their lives suffering from depression, and they are frequently misdiagnosed as UD (Phillips & Kupfer, 2013; Rai et al., 2021). Identifying robust and reliable neural biomarkers for BD is a promising approach for better understanding the underlying neurobiology and improving patient treatment outcomes (Ching et al., 2022; Reavis et al., 2020).

Diverse BD symptoms involve widespread activation across brain regions, and a network‐based investigation may more comprehensively contribute to understanding the pathology of this disorder (Yoon et al., 2021). Neuroimaging studies have implicated dysfunction in emotion regulation networks in BD (Strakowski et al., 2012) that has mainly been incorporated into two theories related to brain imbalance. The first common theory is that the imbalance between top‐down control and bottom‐up affective salience leads to recurrent episodes of mania and depression (Houenou et al., 2011). This theory was supported by the evidence that BD patients show hypoactivation in the top‐down control system (including the ventrolateral prefrontal cortex [VLPFC]) and a hyperactive affective salience system (including the amygdala) (Chen et al., 2011; Delvecchio et al., 2012; Houenou et al., 2011). The second theory involves imbalanced information transmission between the left and right hemispheres (Wang et al., 2015). This theory has been supported by the altered area and shape of the corpus callosum (CC), which is the largest white matter structure connecting the left/right cerebral hemispheres (Marlinge et al., 2014). In addition, reduced fractional anisotropy (FA) in the CC and reduced resting‐state functional connectivity (FC) between hemispheres have also been reported in BD patients (Cui et al., 2020; Emsell et al., 2013; Oertel‐Knöchel et al., 2014; Sarrazin et al., 2014; Wang et al., 2008). Moreover, the imbalance between the sensorimotor network and the default mode network (DMN) with opposite variability patterns is directly linked to depressive and manic episodes (Martino et al., 2016), and the imbalance between the DMN and central executive network (CEN) is also correlated with depressive symptom or trait rumination (Kaiser et al., 2015). The above results at the group‐average level reported widespread BD‐related imbalance in the brain. However, given that extensive individual differences exist among BD patients, whether an individual brain with higher deviation from the balance is associated with more severe BD symptoms, or inversely, whether an individual brain closer to a balance corresponds to milder symptoms, needs to be elucidated.

Normal brain functions involve sufficiently segregated processing in specialized systems and effective global integration (Shine, 2019). Functional segregation and integration in brain FC networks have been proven to be reliable biomarkers for diverse cognitive functions (Cohen & D'Esposito, 2016), and abnormalities in these connected networks have also been linked to many brain disorders (Shine, 2019). Recently, a nested‐spectral partition (NSP) method was developed to identify the balance between segregation and integration in brain functional networks (Wang, Liu, et al., 2021). It was found that the balance in the resting state empowers the brain to support diverse cognitive abilities (Wang, Liu, et al., 2021). Since cognitive impairment is a core illness symptom of BD (Cullen et al., 2016), it follows that an imbalance between segregation and integration may relate to cognitive dysfunction and expression of illness (Sporns, 2013). Thus, we hypothesized that the deviation in resting‐state brain networks from balance is associated with BD, and the resting‐state brain network that is closer to being balanced in BD patients corresponds to milder clinical symptoms. However, when considering the diverse symptom manifestations among BD patients, it would naturally be expected that only some symptoms are related to brain network imbalance. Meanwhile, although many studies have identified differential responses of the brain to depression and mania (Phillips & Kupfer, 2013; Tondo et al., 2017), the heterogeneous neural mechanisms related to distinct BD symptoms still require additional investigation.

Therefore, in this work, we focused on the segregation, integration, and balance in brain FC networks in BD patients and their associations with distinct BD symptoms, that is, mood, daydream, energy, and anxiety. Brain FC networks were constructed using resting‐state functional magnetic resonance imaging (fMRI) dataset in BD patients (*n* = 49) and healthy controls (HCs; *n* = 49). We first studied alterations in network segregation and integration related to BD at the global and system scales. Second, we linked the brain network balance to BD symptoms (i.e., anxiety) and proposed that the balance measure is a better signature. Then, we input network features into a machine‐learning model to predict distinct BD symptoms and investigated their heterogeneous system‐level mechanisms. Finally, we focused on attention‐deficit/hyperactivity disorder (ADHD)‐related network features in BD patients and investigated the common and distinct prediction patterns associated with these two diagnoses.

## 2 MATERIALS AND METHODS

### 2.1 Participants

The dataset was extracted from the UCLA Consortium for Neuropsychiatric Phenomics LA5c Study (Bilder et al., 2018). Forty‐nine patients (age: 34.47±8.93; 21 females) with BD were involved. Since the length of the fMRI data affects the brain FC networks (Wang, Liu, et al., 2021), we controlled and maintained the same number for both BD patients and HCs. Specifically, we first fixed the ratio of males to females as in the BD group and then randomly chose the same number of HCs. Forty‐nine sex‐ and age‐matched HCs (age: 32.95±9.32; 21 females) were selected. There was no significant difference between ages in the two groups (two‐sample *t*‐test, *p* = .724). The diagnoses of BD were provided by the Diagnostic and Statistical Manual of Disorders, Fourth Edition‐Text Revision (Bell, 1994), and based on the Structured Clinical Interview for DSM‐IV (First, 1997). Diagnostic symptoms were evaluated by using 31 questions on the SCID‐I (see Table. S1), which were divided into four clinical items including mood, daydream, energy, and anxiety. Furthermore, the ADHD scores for the same BD patients were also evaluated by using 21 questions (see Table. S2). The detailed criteria of diagnosing BD and ADHD are provided in the Supplemental files.

### 2.2 fMRI data processing

Resting‐state fMRI data (repetition time [TR] = 2 s) has 152 time points and lasted for 304 s. More detailed scanning parameters can be found in (Gorgolewski et al., 2017). The fMRI data were preprocessed according to the standard preprocessing protocols in AFNI (http://afni.nimh.nih.gov/afni/) and FSL (http://www.fmrib.ox.ac.uk/fsl/) (Poldrack et al., 2016). A previous study suggested that framewise displacement (FD) exceeding 0.3 mm should be censored (Drysdale et al., 2017). Here, the mean FD in the HC group was 0.137±0.131, and it was 0.188±0.112 in the BD group. The FD values in the BD and HC groups were significantly different (two‐sample *t*‐test, *p* = .015), and thus an analysis of covariance (ANCOVA) was conducted for the group comparison. Slice‐time correction was applied using the middle slice as the reference frame. Data were normalized by using the standard Montreal Neurological Institute (MNI) unified segmentation and spatially smoothed using a Gaussian kernel of 6‐mm with a full‐width at half‐maximum (FWHM) (Poldrack et al., 2016). The fMRI signal was filtered with a bandpass of 0.01 Hz < *f* < 0.1 Hz. Since the global component of the fMRI fluctuations measured during the resting state is tightly coupled with the underlying neural activity, global signal regression in resting‐state fMRI analyses remains controversial and is not universally recommended (Liu et al., 2017). Thus, the global whole‐brain signal was not removed.

### 2.3 Brain functional networks

The brain was parcellated into N=100 regions according to the Schaefer2018‐100Parcels‐7Networks atlas (Schaefer et al., 2018). This atlas has been widely used to study brain disorders (Wang, Zhen, et al., 2022). The Pearson correlation coefficient between the time series of two regions was calculated to measure FC. The static FC network for each individual was constructed using whole fMRI time series, and the stable FC network was obtained by concatenating all‐time series across all participants in each group. Here, the mean percentage of positive connectivity in individual FC matrices was 93.68% in the HC group and 92.9% in the BD group. In accordance with previous studies (Shappell et al., 2021; Wang, Liu, et al., 2021), negative connectivity was excluded to be zero, and the diagonal elements of the FC matrices were fixed at one. Then, the FC matrix C was decomposed into $C=U\Lambda U^{T}$ (eigenvectors U and eigenvalues Λ), and the eigenmodes were reordered in descending order of Λ. A few negative eigenvalues were set to zero.

### 2.4 Segregation, integration, and balance in brain FC networks

To calculate segregation and integration, the NSP method was first applied to detect the hierarchical modules in brain FC networks with the following procedures (Wang, Liu, et al., 2021):

1. In the first mode, all regions in the eigenvector had the same sign, which is mathematically guaranteed by the Perron–Frobenius theorem. This mode was regarded as the first level with one module (whole‐brain network).
2. In the second mode, the regions with positive eigenvector signs were composited into a module, and the regions with negative signs were assigned as the second module, which was regarded as the second level contrasting two modules.
3. Every module in the second level was further divided into two submodules based on the negative or positive eigenvector signs of regions in the third mode. Consecutively, the FC networks can be modularly partitioned into multiple levels with the order of functional modes increasing until a given level is obtained, wherein each module comprises only a single region (see Figure S1 for a more detailed process).

After each dividing step, the regions were reordered, and the order within modules remained random. During this nested partitioning process, the modular number $M_{i}\left(i=1,\cdots ,N\right)$ and the modular size $m_{j}\left(j=1,\cdots ,M_{i}\right)$ in each level were obtained. Then, at each level, the segregation between the modules and the integration within the modules can be measured by using the following equation:

(1) $$H_{i}=\frac{\Lambda_{i}^{2}M_{i}\left(1-p_{i}\right)}{N}.$$

Here, N normalizes the modular number M to the range (0, 1). $p_{i}=\sum_{j}\left|m_{j}-N/M_{i}\right|/N\;$ is a modular size correction factor that reflects the deviation from the optimized modular size in the *i*th level. Since the first level contains only a single module for the whole FC network, this level is responsible for the global integration:

(2) $$H_{\text{In}}=\frac{H_{1}}{N},$$

and the segregation component is calculated from the second to *N*th levels:

(3) $$H_{\mathrm{Se}}=\sum_{i=2}^{N}\frac{H_{i}}{N}.$$

Here, a higher $H_{\text{In}}$ or smaller $H_{\mathrm{Se}}$ reflects stronger network integration. When the segregation component equals the integration component, the brain FC network is in a balanced state, characterized by an $H_{B}=H_{\text{In}}-H_{\mathrm{Se}}$ close to zero.

Based on the orthogonal and standard eigenvectors, the network integration and segregation components can be mapped to each region *j*:

(4) $$H_{\text{In}}^{j}=H_{1}U_{1j}^{2}\;\text{and}\;H_{\mathrm{Se}}^{j}=\sum_{i=2}^{N}H_{i}U_{\mathrm{ij}}^{2},$$

where $U_{\mathrm{ij}}$ is the eigenvector value for the *j‐*th region at the *i‐*th level. Then, the measure of balance in each region was defined as follows:

(5) $$H_{B}^{j}=H_{\text{In}}^{j}-H_{\mathrm{Se}}^{j},$$

which reflects the balanced contribution of the region to network segregation and integration.

### 2.5 fMRI‐length calibration process

For individual FC networks, a shorter length of the available fMRI time series results in a stronger segregation component (Wang, Liu, et al., 2021). To overcome this limitation, the mean values of segregation and integration components in static FC networks were calibrated to the corresponding segregation and integration components of the stable FC network in each group, that is, $H_{\mathrm{Se}}$ and $H_{\text{In}}$ (Wang, Liu, et al., 2021). In each group, the vectors of segregation (or integration) components of static FC networks for 49 individuals were $H_{\mathrm{Se}}^{S^{\prime }}=\left[H_{\mathrm{Se}}^{1},H_{\mathrm{Se}}^{2},\cdots ,H_{\mathrm{Se}}^{49}\right]$ and $H_{\text{In}}^{S^{\prime }}=\left[H_{\text{In}}^{1},H_{\text{In}}^{2},\cdots ,H_{\text{In}}^{49}\right]$. The corresponding calibrated results were $H_{\mathrm{Se}}^{S}\left(n\right)=H_{\mathrm{Se}}^{S^{'}}\times H_{\mathrm{Se}}/\bar{H_{\mathrm{Se}}^{S^{'}}}$ and $H_{\text{In}}^{S}\left(n\right)=H_{\text{In}}^{S^{'}}\times H_{\text{In}}/\bar{H_{\text{In}}^{S^{'}}}$ for the *n*‐th participant. Here, $\bar{H_{\text{In}}^{S^{\prime }}}$ and $\bar{H_{\mathrm{Se}}^{S^{\prime }}}$ represent the group averages across all individuals, and $H_{\mathrm{Se}}^{S}$ and $H_{\text{In}}^{S}$ represent the calibration, segregation and integration components of the static networks. The regional segregation and integration components were also calibrated in each individual. For region *j* of the *n‐*th individual, the calibrated segregation and integration components were $H_{\mathrm{Se}}^{j}=H_{\mathrm{Se}}^{j^{\prime }}/\bar{H_{\mathrm{Se}}^{S^{'}}}\times H_{\mathrm{Se}}^{S}\left(n\right)$ and $H_{\text{In}}^{j}=H_{\text{In}}^{j^{\prime }}/\bar{H_{\text{In}}^{S^{\prime }}}\times H_{\text{In}}^{S}\left(n\right)$, where the relative contribution of each region to network segregation/integration remained consistent. This calibration has been found to be effective in highlighting the relationships between brain and cognitive abilities (Wang, Zhen, et al., 2022; Wang, Su, et al., 2021).

### 2.6 Machine‐learning approach

The machine‐learning prediction models were constructed using the *scikit‐learn* toolbox (Pedregosa et al., 2011). First, the multivariate linear regression model was built with the function *linear_model. LinearRegression*, wherein independent variable (*x*) is the regional measure, and dependent variable (*y*) is the diagnostic score. Then, leave‐one‐out cross validation (LOO‐CV) was applied with the function *cross_val_predict*. In each iteration of LOO‐CV, one individual was set as the test data set, and the remaining individuals were assigned to training data set. We repeated the process until all individuals were selected as a test data set once. In the prediction model, the features were selected with the functions *f_regression* and *SelectKBest*. The correlation between regional measures and the diagnostic score was first calculated (*f_regression* function), and regions were sorted based on F values. Then, the first K features were selected and input into the prediction model. Herein, we varied K from 1 to *N* and chose the best *K* based on the largest correlation between the real and predicted scores. To test the statistical significance of the predictions (i.e., the *p*‐value), we fixed the selected features and randomized the clinical score (10,000 times). All of the features were normalized, and the weights of the regions were comparable.

## 3 RESULTS

### 3.1 Brain network deviation toward higher segregation in BD patients

**FIGURE 1:**
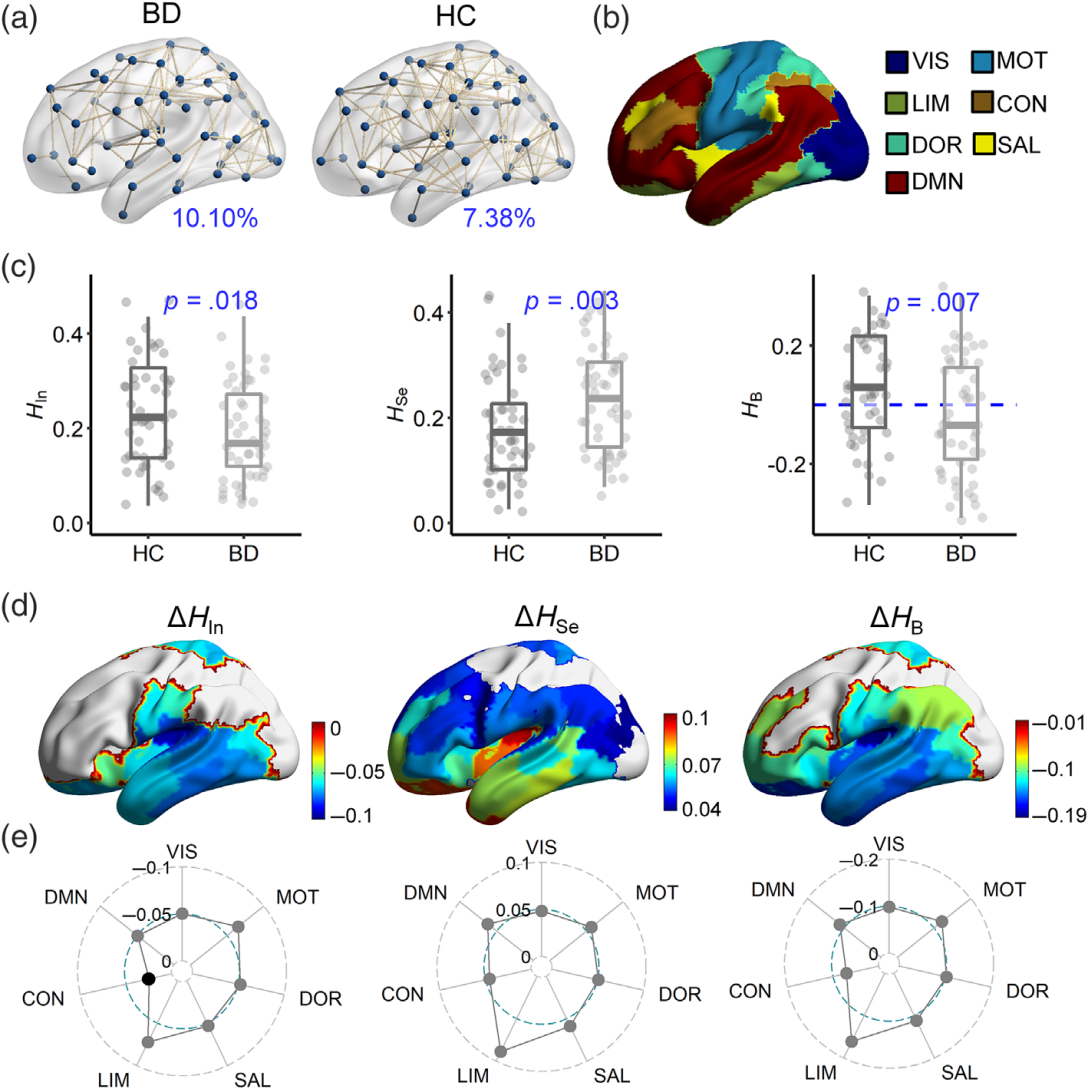
Brain network deviation toward higher segregation in BD patients. (a) Group‐averaged FC network for HC and BD groups, visualized using BrainNet viewer (Xia et al., 2013) with a binarizing threshold of 0.62. The connectivity densities were also provided (*p* = .176; see figure S2 for more comparisons). (b) The partition of seven functional systems. (c) Comparisons of network integration component $H_{\text{In}}$, segregation component $H_{\mathrm{Se}}$, and balance indicator $H_{B}$. (d) Regions with significantly changed $H_{\text{In}}^{i}$, $H_{\mathrm{Se}}^{i}$ and $H_{B}^{i}$ (*p* < .05, uncorrected). The color bar represents the difference between the BD and HC groups. (e) Differences in $H_{\text{In}}$, $H_{\mathrm{Se}}$ and $H_{B}$ in different systems between the HC and BD groups (black dot: *p* > .05).

The group‐averaged FC network in the BD patients had lower connectivity density than that in the HCs (Figure 1a), indicating more segregated brain networks in the resting state. This phenomenon was further supported by the significantly decreased network integration component $H_{\text{In}}$ (ANCOVA, *p* = .018) and increased segregation component $H_{\mathrm{Se}}$ (*p* = .003) in the BD patients (Figure 1c). Meanwhile, by considering the balance between integration and segregation, we found a significant deviation in resting‐state brains toward a more segregated state in the BD patients (*p* = .007; Figure 1c). At the local scale, the regions with significantly increased $H_{\mathrm{Se}}^{i}$ or decreased $H_{\text{In}}^{i}$ and $H_{B}^{i}$ were mainly located in the limbic and motor systems (all *p* < .05, uncorrected; Figure 1b and Figure 1d). Thus, we partitioned the brain into seven functional systems: DMN, control, dorsal attention, salient attention, limbic, motor, and visual systems (Thomas Yeo et al., 2011). All of the functional systems in BD patients had a significant increase in $H_{\mathrm{Se}}$ and a decrease in $H_{\text{In}}$ and $H_{B}$ (all *p* < .05; Figure 1e), with the exception of the control system, which exhibited a difference in $H_{\text{In}}$ that was nonsignificant (*p =* .116), thus indicating higher network segregation in BD patients on the local scale. Moreover, the limbic system exhibited the largest alteration in all of the measures (Figure 1e), followed by the motor system. Thus, the deviation in the brain network toward higher segregation in BD patients is mostly modulated by the limbic and motor systems.

### 3.2 Brain network balance and lower anxiety in BD patients

**FIGURE 2:**
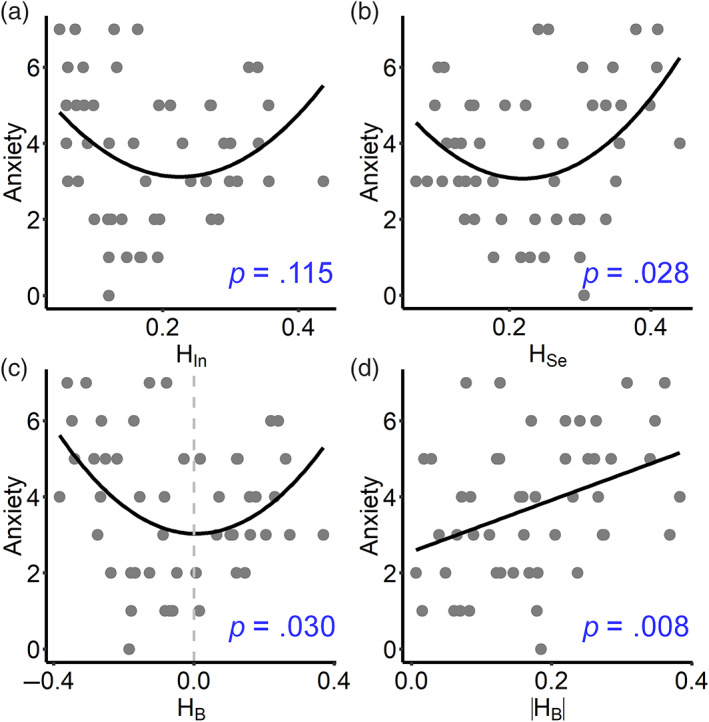
Brain network balance is associated with lower anxiety in BD patients. A quadratic regression model (i.e., *y* ~ *x* ^2^ + *x*) was used to identify relationships between anxiety scores and (a) integration component $H_{\text{In}}$, (b) segregation component $H_{\mathrm{Se}}$ and (c) balance indicator $H_{B}$. (d) Linear relationship between $\left|H_{B}\right|$ and anxiety scores.

We next tested whether the resting‐state brain networks were associated with BD symptoms, including mood, energy, daydreaming, and anxiety. All of the brain network measures ($H_{\text{In}}$, $H_{\mathrm{Se}}$ and $H_{B}$) were not linearly related to any symptom scores in the whole‐brain and system scales (all *p* > 0.05, Table S3). By using the likelihood ratio test (LRT), we found that the quadratic regression model better fit the relationships between network measures and the anxiety score (see Table S4). The anxiety score was nonlinearly related to the segregation component $H_{\mathrm{Se}}$ on the global scale (*p* = .028; Figure 2b) and in the dorsal attention and salient attention systems (see Table S5). The integration component $H_{\text{In}}$ was not significantly related to the anxiety score with a quadratic method (*p* = .115; Figure 2a), but we found a significant quadratic relationship with the salient attention system (*p* = .044; Table S5). Other clinical scores were not significantly related to the segregation and integration components on the global and local scales (see Figure S3). Furthermore, brain FC networks with intermediate levels of segregation and integration were associated with lower anxiety scores, which indicated that a balance between segregation and integration may correspond to lower anxiety in BD patients. Indeed, anxiety scores were significantly related to $H_{B}$ (*p* = .030; Figure 2c), and the *U*‐shaped fitting line suggested lower anxiety for $H_{B}$ near zero. Thus, brain functional networks close to the balance of segregation and integration were associated with milder anxious symptoms in BD patients.

### 3.3 Balance‐based signature predicting BD symptom scores

To further link the brain to distinct BD symptoms, we utilized a machine‐learning approach to predict the symptom scores. As $H_{B}$ has a quadratic relationship with anxiety scores, we linearized it by using the absolute $H_{B}$ that measures the deviation in the brain network from the segregation–integration balance. A higher $\left|H_{B}\right|$ indicates higher deviation from the balance, and there was a nonsignificant difference in $\left|H_{B}\right|$ between the BD and HC groups (*p* = .495; see Figure S4). $\left|H_{B}\right|$ was positively correlated with anxiety scores (*p* = .008; Figure 2d) but was not significantly related to other symptoms (see Table S3).

**FIGURE 3:**
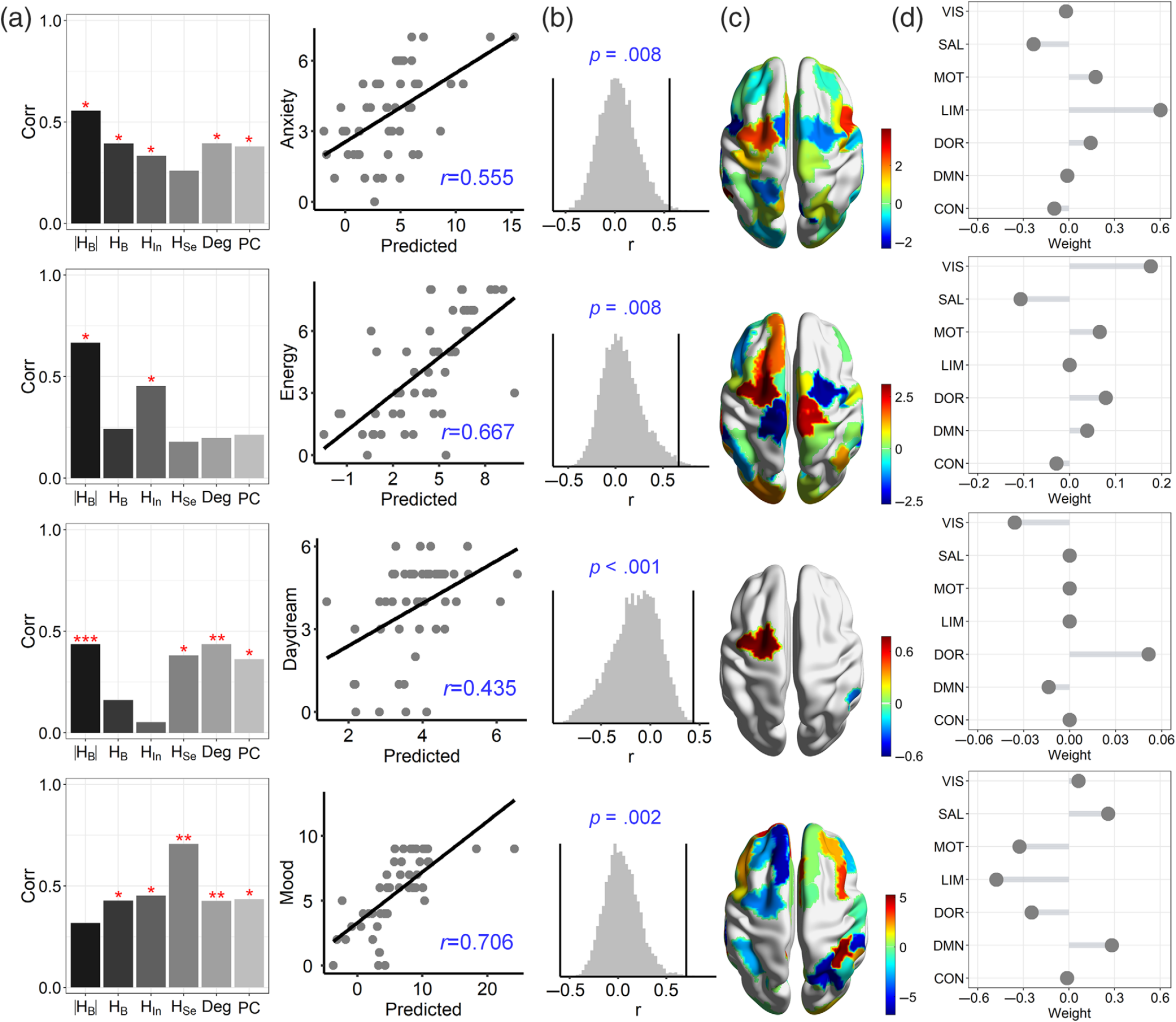
Balanced segregation and integration better predict BD symptoms. (a) Correlations between symptom scores and predicted scores using different brain measures for anxiety, energy, daydream, and mood. As comparisons, the results for degree (Deg; sum of weighted FC) and participant coefficient (PC) were also provided. (b) The best predictions for BD symptoms were statistically tested by using the permutation test (10,000 times). *p < .05, **p < .01, ***p < .001. (c) The weights of regions in the best prediction model were mapped to the brain surface. (d) The mean weight of the regions in systems in the best predictions.

In the machine‐learning prediction model, the input features were regional measures, including $H_{\text{In}}^{i}$, $H_{\mathrm{Se}}^{i}$, $H_{B}^{i}$ and $\left|H_{B}^{i}\right|$. These features were input into multiple linear regression models to perform the predictions, which were further validated by LOO‐CV. In each model, the features were selected from among 100 regions to ensure the best prediction that is measured by the largest correlation between real and predicted scores. As expected, $\left|H_{B}\right|$ better predicted anxiety scores than other brain measures (*r* = 0.555, *p* = .008; Figure 3a, top panel). This better prediction was also maintained for the daydream (*r* = 0.435, *p* < .001) and energy (*r* = .667, *p* = .008) scores, but $H_{\mathrm{Se}}$ better predicted mood (*r* = 0.706, *p* = .002); these data indicated that $\left|H_{B}\right|$ was a fine signature for most BD symptoms except for mood. Meanwhile, the NSP‐based network measures generally had better prediction performance than the classic graph measures on brain network segregation and integration (e.g., node degree and participant coefficient; see Figure 3a), providing further support that NSP‐based analysis is better in linking brain to clinical symptoms (Wang, Zhen, et al., 2022).

Since the features of regions were normalized in the prediction model, the corresponding weights were comparable. In the model, regions with positive weights predicted higher symptom scores, and those with negative weights predicted lower scores. We averaged the weights of regions belonging to the same system to identify the contribution of a particular system to BD symptoms. For anxiety, the limbic system exhibited the highest positive weight, and the salient attention system exhibited a higher negative weight (Figure 3d), thus indicating that higher deviation from the balance in the limbic system or lower deviation in the salient attention system corresponded to higher anxiety. When regarding energy, the visual system exhibited the highest positive weight, and the salient system exhibited a high negative weight, thus suggesting that higher deviation in the salient system or lower deviation in the visual system corresponded to lower energy. Regarding daydream, the dorsal attention system had a high positive weight, and the visual system had the largest negative weight. Thus, a higher deviation from the balance in the visual system or a smaller deviation in the dorsal attention system corresponded to lower daydream scores. Finally, mood was better predicted by $H_{\mathrm{Se}}$, the limbic and motor systems exhibited high negative weights for predicting mood scores and the salient attention and DMN systems exhibited large positive weights, thus indicating that higher integration in the DMN and salient attention systems or larger segregation in the limbic and motor systems that contributed to lower mood. These results offer heterogeneous associations of functional systems with distinct BD symptoms.

### 3.4 Predicting ADHD symptoms in BD patients

The symptomatology of BD and ADHD show high levels of overlap, particularly in adults, and ADHD may cooccur in 25% of BD subjects (Pinna et al., 2019). Crucially, BD patients had a 6.7‐fold higher risk for cooccurring ADHD than the US general population (Merikangas et al., 2007). We thus next studied whether ADHD and BD scores were associated with common and distinct networks. This investigation can be helpful for more accurately diagnosing ADHD in BD patients, and it has been suggested that such screening procedures should be common in the current BD diagnosis process (Halmøy et al., 2010).

**FIGURE 4:**
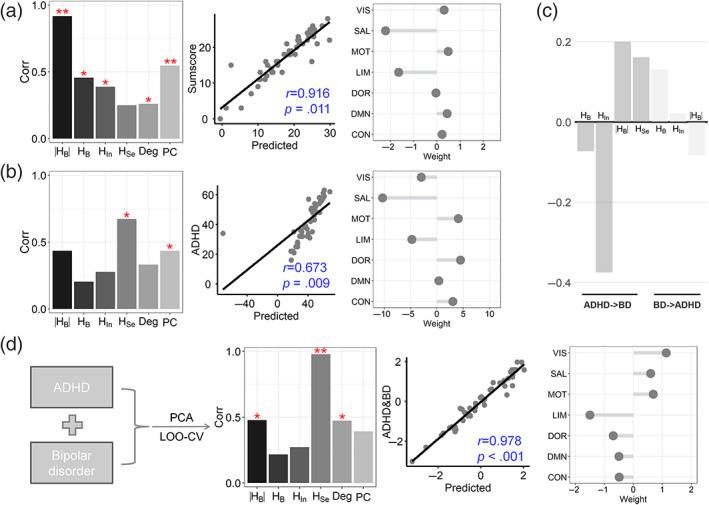
Predictions of BD and ADHD scores. (a) Predictions of BD sumscores. From the left panel to the right panel: Prediction performances, the best prediction, and the weights in seven systems. (b) Predictions of ADHD scores. (c) he selected features in (a) were used to predict ADHD scores, and the features in (b) were used to predict BD scores. (d) PCA was applied to obtain the dominant common score, which was subsequently predicted through the machine‐learning approach.

None of the brain measures were linearly or quadratically correlated with ADHD scores (see Figure S5) or BD sumscores; thus, we utilized the machine‐learning approach to perform the prediction. Due to the fact that BD sumscores involved nonlinearity in relation to anxiety, it is expected that the BD sumscores could be best predicted by the $\left|H_{B}\right|$ (*r* = 0.916, *p* = .011) relative to other brain measures (Figure 4a). A higher deviation from the balance in the salient attention and limbic systems and a lower deviation in the DMN and motor systems contributed to smaller BD sumscores (Figure 4a). ADHD scores were best predicted by $H_{\mathrm{Se}}$ (*r* = 0.673, *p* = .009); in addition, the motor and dorsal attention systems exhibited high positive weights, and the salient attention and limbic systems exhibited high positive weights (Figure 4b). Moreover, the features based on the NSP method generally exhibited better predictions than the classic graph analysis. Thus, it seems that the BD and ADHD scores share abnormalities in the salient attention, limbic, and motor systems, but it was unclear whether the features that were identified in the ADHD prediction model can also predict the BD score, or vice versa. We found that the selected features (i.e., $H_{B}$, $H_{\text{In}}$, $\left|H_{B}\right|$) in the BD prediction models could not predict ADHD scores, and the selected features (i.e., $H_{B}$, $H_{\text{In}}$, $\left|H_{B}\right|$ and $H_{\mathrm{Se}}$) in the ADHD prediction models could not predict BD sumscores (Figure 4c). Thus, these prediction models for BD and ADHD scores are specific and distinct from each other, which has great potential application for the screening of ADHD in BD patients.

Notably, the diagnostic items for BD and ADHD highly overlap, which is also reflected in the high positive correlation between BD and ADHD scores (*r* = 0.456, *p* = .001; see Figure S6). We applied a principal component analysis (PCA) to the BD and ADHD scores and extracted the first component (which explained 72.8% of the variance), which we viewed as the common score contributed by shared symptoms between BD and ADHD, such as mood instability, impulsivity, and impatience (O'Connell et al., 2021). Then, we used the machine‐learning approach to predict this common score. This operation contributes to the investigation of the neural mechanisms underlying the overlapped symptoms between BD and ADHD. $H_{\mathrm{Se}}$ best predicted the common score (*r* = 0.978, *p* < .001; Figure 4d). The limbic system exhibited a high negative weight, and the visual and motor systems exhibited high positive weights. Thus, higher levels of segregation in the visual and motor systems, as well as a higher level of integration in the limbic system, contributed to higher common scores for ADHD and BD.

## 4 DISCUSSION

To link aspects of the brain with BD clinical symptoms, we measured the functional segregation, integration, and balance across hierarchical modules in resting‐state brain FC networks. We found that resting‐state brain networks deviated toward more segregation in BD patients relative to HCs and that the limbic system was the most sensitive to BD. Then, we provided the first evidence that BD patients with a balance between segregation and integration in brain functional networks have milder anxiety symptoms. Using a machine‐learning approach, we suggested that compared to classic graph analysis, the NSP‐based balance indicator can better predict most BD symptoms, with the exception of mood, and we also identified heterogeneous associations between distinct symptoms and functional systems. Finally, we specifically predicted ADHD and BD scores, as well as their common domains. These results support several basic hypotheses regarding BD, reveal the distinct neural bases of BD symptoms, and contribute to the screening of ADHD in BD patients.

### 4.1 High deviation in brain functional networks toward more segregation in BD patients

BD is believed to be associated with brain imbalance in terms of multiple aspects, including top‐down control versus bottom‐up affective salience (Chen et al., 2011; Delvecchio et al., 2012; Houenou et al., 2011), left hemisphere versus right hemisphere (Emsell et al., 2013; Oertel‐Knöchel et al., 2014; Sarrazin et al., 2014; Wang et al., 2008), sensorimotor network versus DMN (Martino et al., 2016), and DMN versus CEN (Kaiser et al., 2015). Normal brains in the resting state are expected to maintain a functional balance between segregation and integration that allows highly efficient communication on local and global scales and relates to the best memory ability (Wang, Goerlich, et al., 2021). Thus, an imbalance in the segregation and integration in brain functional networks is expected to be associated with brain disorders, including BD. Based on graph theory methods, previous studies have consistently obtained higher segregation in BD patients, such as increased characteristic path length, decreased global efficiency, and decreased rich‐club and feeder connectivity density (Doucet et al., 2017; Nogovitsyn et al., 2020; O'Donoghue et al., 2017; Vandekerckhove et al., 2020; Wang et al., 2017; Wang, Lin, et al., 2019). Here, we found that the resting brain state of BD patients deviated from a balanced state toward more segregation, providing further evidence that BD is related to brain imbalance. Meanwhile, we also found that the limbic system showed the largest alterations in BD patients. This system involves a set of regions in the paleocortex and supports a variety of functions related to emotion regulation and motivation meditation (Dalgleish, 2004; Price and Drevets, 2010). Previous studies have found altered FC in the limbic network in BD patients (Chen et al., 2018; Wang et al., 2016; Wessa et al., 2014), and structural neuroimaging investigations have reported volumetric disturbances in limbic areas (Price & Drevets, 2010; Williams et al., 2021; Zhigalov et al., 2017). In addition, a direct correlation of the limbic network to emotional dysregulation in BD patients has also been observed (Blond et al., 2012; Wessa et al., 2014). Herein, by using a machine‐learning approach, we further found that the limbic system dominantly predicted anxiety and mood symptoms, which are both crucially related to emotional dysfunction. This result provides further support for the notion that BD is related to dysfunction in network related to emotion regulation (Strakowski et al., 2012).

### 4.2 Associations between anxiety and brain imbalance in BD patients

BD is associated with large individual differences in clinical symptoms and brain network changes. The mechanisms underlying the imbalance in BD suggest that individuals with a high deviation from a balanced system may have higher symptom scores. Using the NSP method, we provided the first evidence that the balanced brain corresponds to lower anxiety in individual BD patients, consistent with the imbalance theory regarding BD at the group level. Anxiety is highly relevant to human health, and in BD patients, it has also been suggested to be a risk factor for negative outcomes (De Berardis et al., 2017). Thus, identifying neurobiological markers signaling anxiety is important for assessing individuals with a high risk for mental illness. In euthymic BD patients, an increase in high‐frequency spectral content in the DMN was associated with higher levels of anxiety symptoms (Marino et al., 2021), but in a FC analysis, no significant correlation with anxiety was found (Rey et al., 2016). Apparently, few studies have successfully linked aspects of the brain to anxiety in BD patients, and the mechanisms underlying anxiety are poorly understood. Our results suggested that this limitation may be due to the nonlinear relationship between brain networks and anxiety, which was possibly overlooked in previous studies. In HCs, connectome‐based predictive modeling (CPM) predicted the degree of trait anxiety and found anxiety‐related FC between limbic areas and the prefrontal cortex in the DMN (Wang, Su, et al., 2021). In addition, in healthy adults across the lifespan, anxiety‐related FC has been found between limbic‐motor systems and between limbic‐dorsal attention systems (He et al., 2015). Herein, we also found that in BD patients, a higher deviation from the balance in the limbic system predicts severe anxiety. Our results suggest that the direct modulation of an abnormal limbic system may be the neural mechanism underlying anxiety in BD patients.

### 4.3 Daydreaming and energy symptoms in BD patients

Daydreaming represents a consciousness state between dreaming and waking cognition that is stimulus‐independent and task‐unrelated (Poerio et al., 2016). Excessive daydreaming has been associated with vulnerability and risk for mania and can predict a switch from UD to BD (Akiskal et al., 1995). We found that the dorsal attention and visual systems are highly involved in the prediction of daydreaming in BD patients, and these two systems have opposite correlations. The dorsal attention system is made up of the frontal eye fields and inferior parietal sulcus (Owens et al., 2019). Using dynamic causality analysis, the dorsal attention system was found to exert top‐down influences on visual areas during the spatial orienting of attention, which was greater than the reverse bottom‐up effects from the visual cortex (Bressler et al., 2008; Roebroeck et al., 2005). Thus, the opposite effects associated with daydreaming may suggest abnormal pathways between these two systems that are incapable of effectively suppressing attention to stimulus‐independent thoughts. Meanwhile, the DMN has been widely observed to participate in both daydreaming and dreaming (Buckner et al., 2008), and individual differences in daydreaming in daily life were also associated with the DMN (Kucyi & Davis, 2014). However, the DMN was not predominantly important in the prediction model of daydreaming in the BD patients. The dynamic cooperation between the DMN and visual system was found to correlate with daydreaming (Kucyi & Davis, 2014), and the abnormal interaction between the DMN and dorsal attention system has been related to sleep deprivation (De Havas et al., 2012) and numerous neurological and psychiatric disorders (Owens et al., 2019). Thus, our results may indicate that the DMN, by modulating the opposite functional roles between visual and dorsal attention systems, indirectly participates in excessive daydreaming in BD patients.

Similar to daydreaming, energy has been regarded as a central symptom of BD, and “increased energy or activity” has been added to criterion A in the DSM‐5 (Association, 2013). Increased energy is associated with mania in BD, and decreased energy is related to depression (Cheniaux et al., 2017). However, few studies have investigated the mechanisms underlying this form of energy. Using a multiple kernel learning (MKL) approach, Oliveira et al found that the VLPFC made the highest contribution to the prediction of energy‐manic symptoms (de Oliveira et al., 2019). Herein, in the machine‐learning prediction framework, the salient attention system (including part of the VLPFC) had the highest negative contribution to energy, and the visual system had the highest positive contribution. The salient attention system plays a key role in detecting and segregating important information from insignificant stimuli. Abnormalities in this system may be related to the brain imbalance between top‐down and bottom‐up visual information processing (Maekawa et al., 2013) Thus, our results may indicate incongruous cooperation among salient attention and visual systems in BD patients.

### 4.4 ADHD symptoms in BD patients

For BD patients, the evaluation of symptom severity is important in the clinic. When combining the NSP method and a simple machine‐learning model, we found that the BD sumscore can be predicted with excellent precision (by a correlation up to 0.916), primarily because we considered the corresponding nonlinearity. The classical graph theory measures (e.g., degree and participant coefficient) are incapable of identifying the balance and have considerably worsened prediction performance than the NSP‐based analysis method. This result is highly consistent with previous results that demonstrated that the NSP‐based method is better in linking brain networks to cognitive abilities (Wang, Liu, et al., 2021), task behavior (Wang, Su, et al., 2021), ADHD symptoms (Wang, Fan, et al.,  2022) and stress conditions (Wang, Zhen, et al., 2022). More importantly, the excellent prediction on the BD sumscore provides a potentially useful application of the NSP‐based framework to accurately diagnose BD in a timely manner and to improve medical treatment.

BD and ADHD have many overlapping clinical symptoms (O'Connell et al., 2021), and screening ADHD is important for the appropriate treatment of BD (Halmøy et al., 2010). In general, the common and distinct alterations in brain networks in ADHD and BD patients, relative to HCs, have been widely documented (Passarotti et al., 2010). For example, compared to HCs, BD is related to higher segregation, and ADHD is associated with higher integration (Wang, Fan, et al., 2022). In the comorbid state of ADHD plus BD, Biederman et al suggested that ADHD and BD independently contribute to volumetric alterations in the brain (Biederman et al., 2008), and the impulsivity that is common to ADHD and BD may have different neural mechanisms mediated by reward systems (Passarotti & Pavuluri, 2011). Herein, BD patients had ADHD scores below the criterion, but we also found similar prediction patterns for ADHD and BD scores, which involved the motor, salient attention, and limbic systems. The motor system is responsible for brain activation during task performance, and abnormal communication with other systems was suggested to underlie the depressive and manic phases of BD (Martino et al., 2016), as well as the hyperactivity of ADHD (Gilbert et al., 2019). Furthermore, less segregation in the limbic and salient attention systems contributed to higher ADHD scores, which is consistent with a previous study that higher integration in the limbic system better predicts severe hyperactivity of ADHD, and a higher integration in salient attention system better predicts higher inattention (Wang, Fan, et al., 2022). However, the best prediction models were not shared for ADHD and BD scores, which may be crucially important for distinguishing ADHD from BD, or vice versa. Notably, BD and ADHD scores are highly correlated, and the latent common domain was perfectly predicted by our NSP‐based measure. Those signatures were distributed in the limbic and visual systems that have been widely reported in ADHD and BD studies (Ahrendts et al., 2011; Maekawa et al., 2013; Wang, Fan, et al., 2022). While these two systems are closely related to hyperactivity/impulsivity and inattention (Ahrendts et al., 2011), our results may explain the neural mechanisms contributing to common symptoms between BD and ADHD, such as impulsivity and impatience (O'Connell et al., 2021).

## 5 LIMITATIONS OF THE STUDY

The sample size of this study may have been a limitation. The relationship with functional balance was only observed for the anxiety symptom. However, the balance‐related measure can also best predict the energy score, and the LRT result indicated a nonlinear relationship between network segregation and mood in the limbic system (*p* = .041, see Table S4). A larger sample size may contribute to identifying clearer nonlinear relationships between brain networks and energy/mood symptoms. Furthermore, our results suggested that NSP‐based measures may be biomarkers for BD, and a larger sample size is also necessary for the application of the NSP‐based framework to classify BD patients from HCs. Finally, although we demonstrated the association between brain networks and BD symptoms, the neural mechanism underlying these symptoms are still unclear. The large‐scale brain modeling is a powerful approach to analyze the dynamic mechanism and has been applied to brain disorders and cognition (Chen et al., 2015; Herzog et al., 2022; Zhang et al., 2021). The main findings that were identified in this study need to be further explained by building a large‐scale brain model of BD patients.

## 6 CONCLUSION

In summary, the hierarchical module analysis enabled the hypothesis‐driven discovery that linked functional imbalance in the brain to anxiety in BD patients and revealed heterogeneous neural mechanisms associated with multiple BD symptoms. The identified network features provide insight into the neurobiological mechanisms supporting common and distinct clinical components of BD and ADHD, which may in turn have implications for the development of more objective and accurate diagnostic standards for BD and contribute to the screening of ADHD in BD patients.

## DECLARATION OF COMPETING INTERESTS

All authors declare no competing interests.

## AUTHOR CONTRIBUTIONS

Zhao Chang: Data Processing, Formal Analysis, Writing‐original draft, Software. Xinrui Wang: Visualization Writing ‐ Review and Editing. Ying Wu: Writing‐Review and Editing. Rong Wang: Methodology, Funding Acquisition, Writing‐Introduction and Discussion, Writing‐Review and Editing. Pan Lin: Writing‐Review and Editing, Funding Acquisition.

## Supporting information


**Appendix S1** Supporting Information.

## Data Availability

The MRI data and clinical scores are available at https://openfmri.org/dataset/ds000030/.The codes are available at https://github.com/TobousRong/bipolar-disorder.
