# Supplementary material for: Segregation, integration and balance in resting‐state brain functional networks associated with bipolar disorder symptoms
Source: Hum Brain Mapp. 2022 Sep 26;44(2):599–611. doi: 10.1002/hbm.26087 (PMC9842930; doi:10.1002/hbm.26087)
Supplement: Supplementary file 1 — Appendix S1 Supporting Information. [file HBM-44-599-s001.docx]

**Table S1** Criteria of diagnosing BD.

| Score Names | Question Description |
| --- | --- |
| Mood | My mood often changes, from happiness to sadness, without my knowing why. |
|  | I have frequent ups and downs in mood, with and without apparent cause. |
|  | I often feel guilty without a very good reason for it. |
|  | My feelings are rather easily hurt. |
|  | There are times when my future looks very dark. |
|  | Ideas run through my head so that I cannot sleep. |
|  | I keep in fairly uniform spirits. |
|  | Often I find it difficult to go to sleep because of thinking what happened during the day. |
|  | I often feel disgruntled. |
| Daydream | I daydream a great deal. |
|  | I like to indulge in a reverie (daydreaming). |
|  | I daydream very little. |
|  | I frequently find myself in a meditative state. |
|  | I am inclined to think about myself much of the time. |
|  | My daydreams are frequently about things that can never come true. |
| energy | I am inclined to rush from one activity to another without pausing for enough rest. |
|  | I am a horse for work; I am seldom exhausted. |
|  | I am the kind of person who is\on the go\all the time. |
|  | I am able to work unusually long hours without feeling tired. |
|  | I am often so much on the go that sooner or later I wear myself out. |
|  | I am quick in my actions. |
|  | I am happiest when I get involved in some project that calls for rapid action. |
|  | I have experienced periods so full of pep that sleep didn't seem to be necessary for days at a time. |
| anxiety | It is hard for me to ask someone for a favor. |
|  | When I meet new people, I am afraid I won't do the right thing. |
|  | I feel that I never really get all that I need from people. |
|  | I don't like to buy clothes for myself. |
|  | I would rather stay free of involvements with others than risk disappointments. |
|  | While in trains, buses, etc. I often talk to strangers. |
|  | Hope only brings disappointment. |
|  | I am inclined to be shy in the presence of the opposite sex. |

**Table S2** Criteria of diagnosing ADHD.

| Score Names | Question Description |
| --- | --- |
| Adult Attention: | Makes a Lot of Careless Mistakes. |
|  | Difficulty Sustaining Attention on Tasks/Play Activities. |
|  | Doesn't Listen. |
|  | Difficulty Following Instructions. |
|  | Difficulty Organizing Tasks. |
|  | Dislikes/Avoids Tasks Requiring Attention. |
|  | Loses Things. |
|  | Easily Distracted. |
|  | Forgetful in Daily Activities. |
| Adult Hyperactivity | Fidget. |
|  | Difficulty Remaining Seated. |
|  | Runs or Climbs Excessively. |
|  | Difficulty Playing Quietly. |
|  | On the Go/Acts Like Driven by Motor. |
|  | Talks Excessively. |
|  | Blurts Out Answers. |
|  | Difficulty Waiting Turn. |
|  | Interrupts or Intrudes. |
| Adult Duration | Duration of childhood symptoms. |
| Adult Impairment | Some impairment in 2 or more settings. |
|  | Assess clinically significant impairment. |

**Table S3** Linear relationships between clinical scores and brain measures in the whole-brain and seven systems. The significant linear relationships were marked by yellow colors.

| Sumscore | | | | | | | | |
| --- | --- | --- | --- | --- | --- | --- | --- | --- |
| corr  (*p*-value) | Whole brain | VIS | MOT | DOR | SAL | LIM | CON | DMN |
| H_In_ | 0.071  (0.630) | 0.063  (0.669) | 0.037  (0.801) | 0.079  (0.591) | 0.038  (0.793) | 0.035  (0.811) | 0.140  (0.339) | 0.073  （0.620） |
| H_Se_ | -0.023  (0.874) | -0.065  (0.656) | -0.000  (0.999) | -0.043  (0.772) | -0.004  (0.979) | 0.033  (0.821) | -0.065  (0.657) | 0.004  (0.979) |
| H_B_ | 0.047  (0.748) | 0.065  (0.658) | 0.020  (0.892) | 0.062  (0.673) | 0.021  (0.885) | -0.005  (0.971) | 0.102  (0.488) | 0.032  (0.826) |
| \|H_B_\| | 0.189  (0.191) | 0.091  (0.535) | 0.197  (0.175) | 0.232  (0.109) | 0.248  (0.085) | 0.060  (0.684) | 0.137  (0.349) | 0.163  (0.263) |
| Mood | | | | | | | | |
| H_In_ | 0.011  (0.940) | 0.002  (0.991) | -0.027  (0.852) | 0.027  (0.854) | -0.017  (0.909) | 0.013  (0.930) | 0.095  (0.516) | 0.005  (0.974) |
| H_Se_ | 0.027  (0.851) | -0.006  (0.967) | 0.041  (0.781) | 0.004  (0.980) | 0.054  (0.713) | 0.058  (0.694) | -0.028  (0.848) | 0.059  (0.688) |
| H_B_ | -0.008  (0.954) | 0.004  (0.979) | -0.034  (0.817) | 0.012  (0.933) | -0.036  (0.807) | -0.030  (0.840) | 0.060  (0.680) | -0.030  (0.840) |
| \|H_B_\| | 0.184  (0.206) | 0.094  (0.520) | 0.140  (0.339) | 0.201  (0.167) | 0.267  (0.063) | 0.075  (0.607) | 0.164  (0.261) | 0.197  (0.175) |
| Energy | | | | | | | | |
| H_In_ | 0.191  (0.188) | 0.169  (0.246) | 0.201  (0.167) | 0.235  (0.104) | 0.186  (0.200) | 0.063  (0.668) | 0.199  (0.171) | 0.160  (0.271) |
| H_Se_ | -0.180  (0.215) | -0.165  (0.256) | -0.178  (0.221) | -0.238  (0.100) | -0.226  (0.118) | -0.068  (0.643) | -0.165  (0.257) | -0.137  (0.347) |
| H_B_ | 0.187  (0.197) | 0.169  (0.244) | 0.192  (0.186) | 0.239  (0.098) | 0.209  (0.150) | 0.068  (0.644) | 0.184  (0.207) | 0.150  (0.303) |
| \|H_B_\| | -0.015  (0.918) | 0.029  (0.844) | 0.103  (0.480) | 0.037  (0.800) | -0.035  (0.814) | -0.056  (0.702) | -0.041  (0.779) | -0.067  (0.649) |
| Daydream | | | | | | | | |
| H_In_ | 0.079  (0.588) | 0.090  (0.541) | 0.070  (0.631) | 0.049  (0.739) | 0.045  (0.759) | 0.075  (0.608) | 0.102  (0.484) | 0.095  (0.516) |
| H_Se_ | -0.059  (0.689) | -0.137  (0.349) | -0.041  (0.782) | -0.043  (0.771) | -0.008  (0.955) | -0.050  (0.733) | -0.060  (0.682) | -0.046  (0.753) |
| H_B_ | 0.070  (0.635) | 0.113  (0.437) | 0.057  (0.697) | 0.046  (0.752) | 0.027  (0.855) | 0.062  (0.672) | 0.081  (0.580) | 0.070  (0.633) |
| \|H_B_\| | 0.035  (0.811) | -0.088  (0.550) | 0.044  (0.764) | 0.087  (0.550) | 0.105  (0.473) | -0.036  (0.804) | 0.010  (0.944) | 0.044  (0.765) |
| Anxiety | | | | | | | | |
| H_In_ | -0.107  (0.464) | -0.099  (0.499) | -0.171  (0.241) | -0.134  (0.357) | -0.138  (0.344) | -0.053  (0.717) | -0.025  (0.864) | -0.063  (0.667) |
| H_Se_ | 0.179  (0.217) | 0.136  (0.352) | 0.220  (0.128) | 0.212  (0.143) | 0.224  (0.123) | 0.169  (0.247) | 0.098  (0.502) | 0.156  (0.285) |
| H_B_ | -0.145  (0.320) | -0.118  (0.419) | -0.196  (0.177) | -0.174  (0.231) | -0.183  (0.208) | -0.125  (0.393) | -0.065  (0.658) | -0.114  (0.435) |
| \|H_B_\| | 0.373  (0.008) | 0.217  (0.134) | 0.294  (0.041) | 0.372  (0.009) | 0.412  (0.003) | 0.205  (0.157) | 0.276  (0.055) | 0.322  (0.024) |

**Table S4** Likelihood ratio test (LRT) between the linear and quadratic regression modes. In LRT, the origin hypothesis H_0_: linear model has a good fit, and the alternative hypothesis H_1_: quadratic model has a better goodness of fit. If the *p*-value of LRT is smaller than 0.05, we rejected the H_0_ hypothesis and chosen the quadratic model, otherwise, the linear regression model was chosen. The quadratic model can better describe the relationship between anxiety score and brain network measures than the linear regression model.

| H_In_ | | | | | | | | |
| --- | --- | --- | --- | --- | --- | --- | --- | --- |
| Lrtest  (p-value) | Whole Brain | VIS | MOT | DAR | SAL | LIM | CON | DMN |
| Sumscore | 0.313 | 0.811 | 0.383 | 0.271 | 0.232 | 0.735 | 0.233 | 0.356 |
| Mood | 0.522 | 0.737 | 0.586 | 0.507 | 0.146 | 0.957 | 0.150 | 0.414 |
| Energy | 0.855 | 0.752 | 0.947 | 0.848 | 0.570 | 0.974 | 0.933 | 0.933 |
| Daydreaming | 0.764 | 0.548 | 0.936 | 0.684 | 0.684 | 0.832 | 0.880 | 0.888 |
| Anxiety | 0.040 | 0.183 | 0.034 | 0.025 | 0.015 | 0.304 | 0.024 | 0.079 |
| H_Se_ | | | | | | | | |
| Lrtest  (p-value) | Whole Brain | VIS | MOT | DAR | SAL | LIM | CON | DMN |
| Sumscore | 0.073 | 0.539 | 0.112 | 0.100 | 0.125 | 0.037 | 0.102 | 0.087 |
| Mood | 0.085 | 0.398 | 0.157 | 0.191 | 0.086 | 0.041 | 0.063 | 0.115 |
| Energy | 0.556 | 0.790 | 0.368 | 0.678 | 0.917 | 0.273 | 0.865 | 0.359 |
| Daydreaming | 0.624 | 0.335 | 0.684 | 0.444 | 0.544 | 0.398 | 0.686 | 0.418 |
| Anxiety | 0.014 | 0.156 | 0.065 | 0.010 | 0.014 | 0.060 | 0.019 | 0.114 |
| H_B_ | | | | | | | | |
| Lrtest  (p-value) | Whole Brain | VIS | MOT | DAR | SAL | LIM | CON | DMN |
| Sumscore | 0.137 | 0.643 | 0.199 | 0.138 | 0.155 | 0.144 | 0.100 | 0.138 |
| Mood | 0.142 | 0.538 | 0.288 | 0.173 | 0.125 | 0.386 | 0.126 | 0.169 |
| Energy | 0.889 | 0.964 | 0.706 | 0.976 | 0.752 | 0.328 | 0.670 | 0.633 |
| Daydreaming | 0.741 | 0.426 | 0.814 | 0.650 | 0.515 | 0.397 | 0.616 | 0.556 |
| Anxiety | 0.011 | 0.131 | 0.027 | 0.006 | 0.010 | 0.098 | 0.018 | 0.056 |

**Table S5** Using the quadratic regression model to identify the nonlinear relationship between anxiety and brain measures in the whole-brain and seven systems (significant relationship was marked by yellow colors, mark: ‘--’ mean that LRT test was insignificant.)

| Anxiety | | | | | | | | |
| --- | --- | --- | --- | --- | --- | --- | --- | --- |
| Nonlinear cor | Whole Brain | VIS | MOT | DAR | SAL | LIM | CON | DMN |
| H_In_ | 0.115 | -- | 0.070 | 0.069 | 0.044 | -- | 0.089 | -- |
| H_Se_ | 0.028 | -- | -- | 0.015 | 0.018 | -- | 0.061 | -- |
| H_B_ | 0.030 | -- | 0.042 | 0.014 | 0.020 | -- | 0.062 | -- |


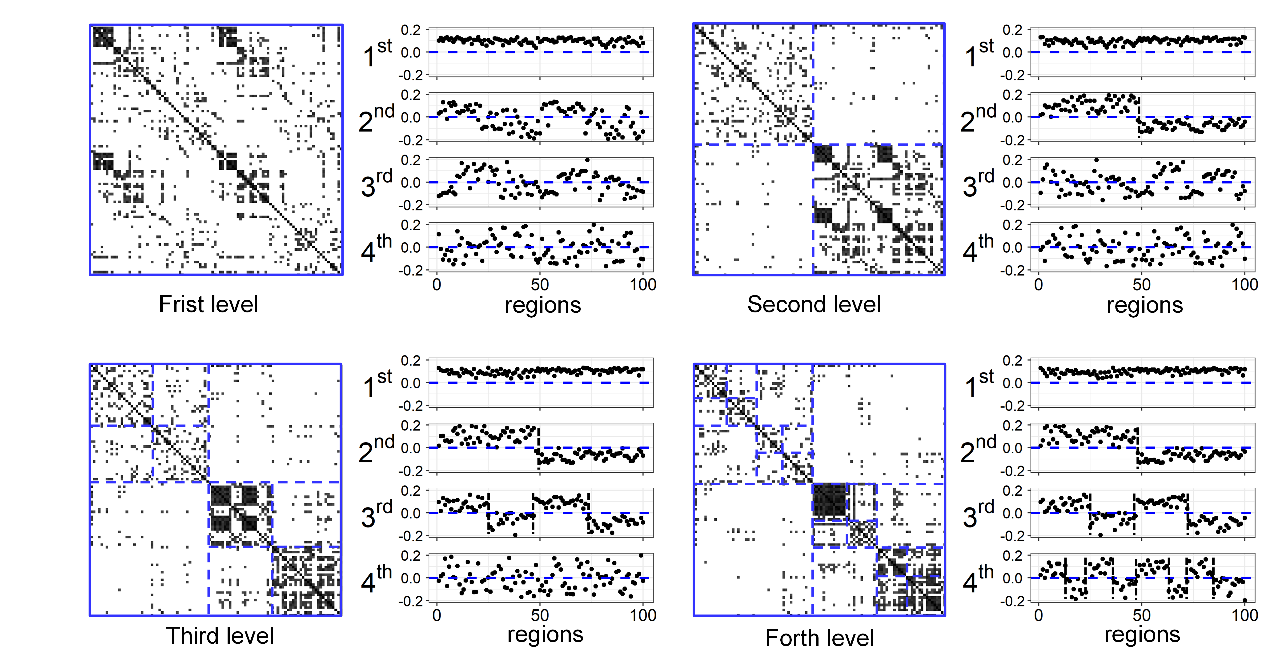


**Fig. S1** Illustration of the partition of the FC network into hierarchical modules in the first four levels. The blue dashed lines represent the boundaries of hierarchical modules suggested by the NSP method. Note that the FC network was binarized for clearer illustration, but the NSP method was performed at weighted networks. A hierarchical modular partition in the first four functional modes was also provided, where modules in each level were detected according to the positivity or negativity of eigenvector components. Note that after each partitioning step, the regions were reordered, and the order within modules remained random.


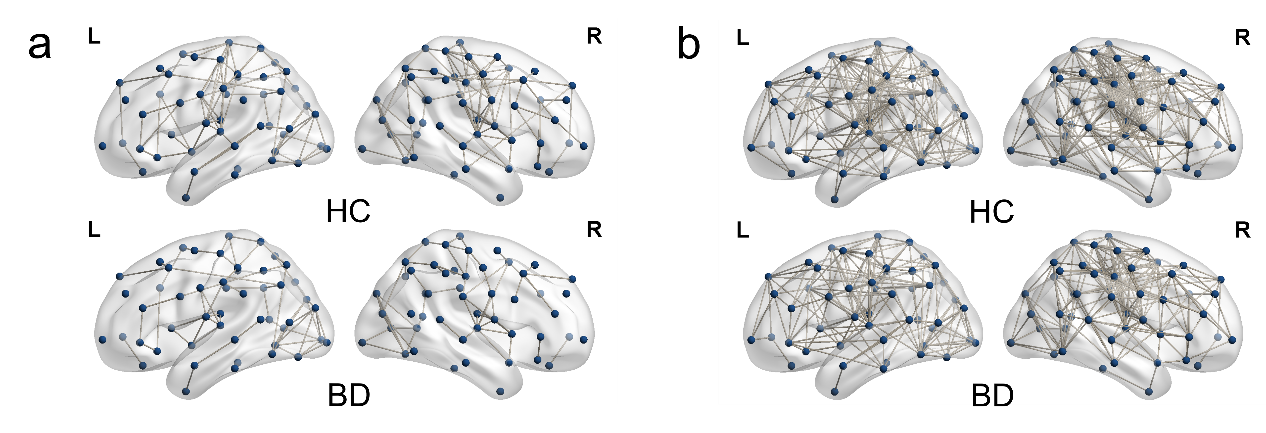


**Fig. S2** Comparison of group-averaged FC networks between HC and BD groups. **(a)** Threshold of 0.67 (*p*=0.154) and **(b)** 0.57 (*p*=0.186)


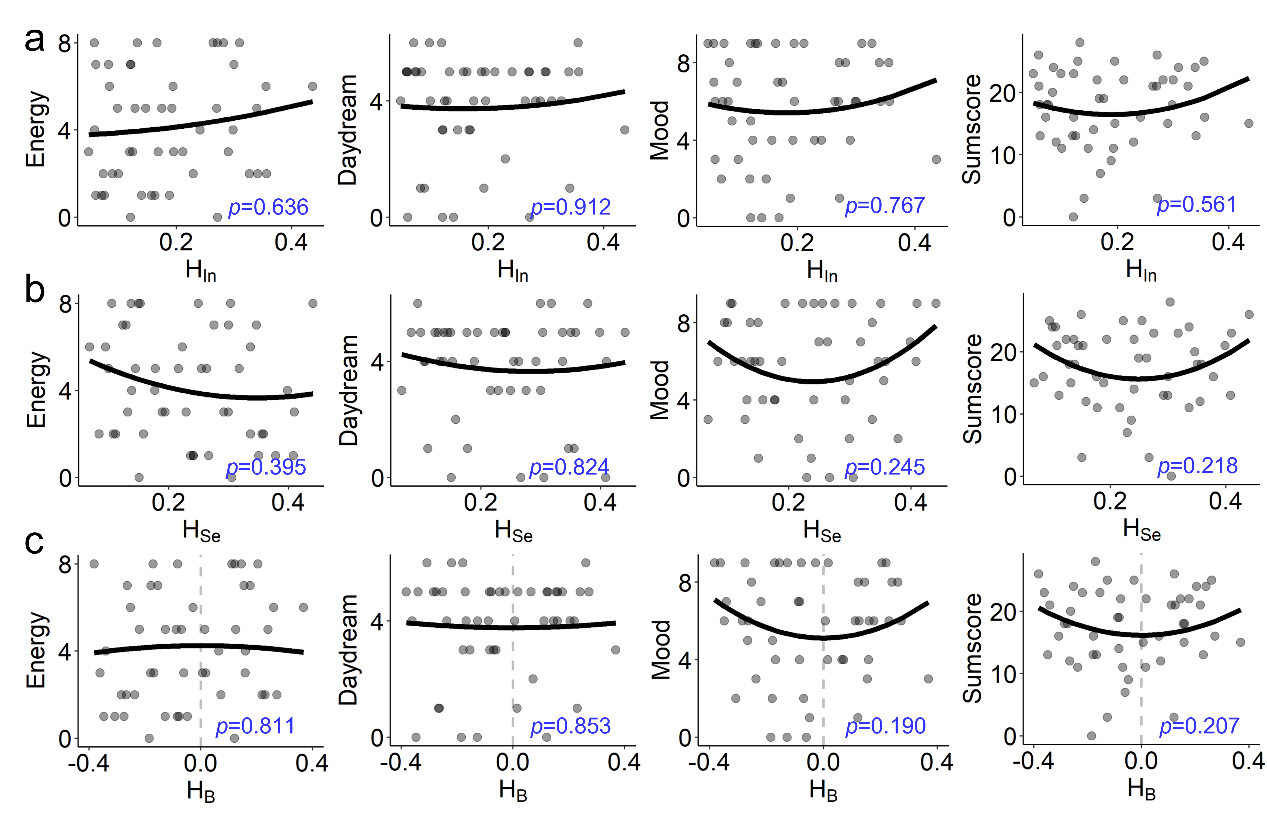


**Fig. S3** Clinical scores except for anxiety were insignificantly related to brain measures in a nonlinear manner. Those fitting lines were obtained using the quadratic regression model.


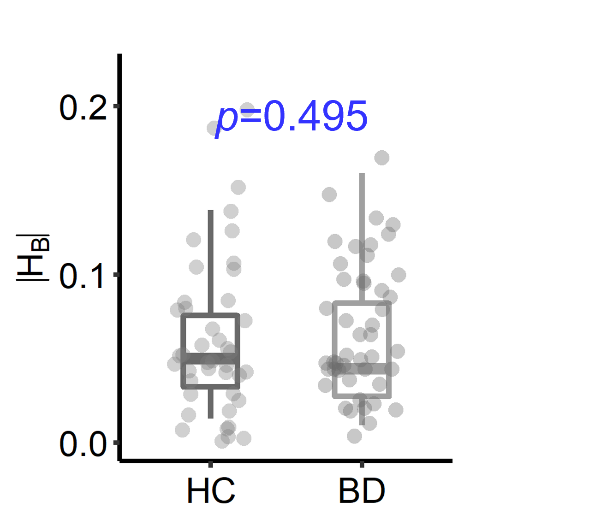


**Fig. S4** |H_B_| was insignificantly different between two groups.


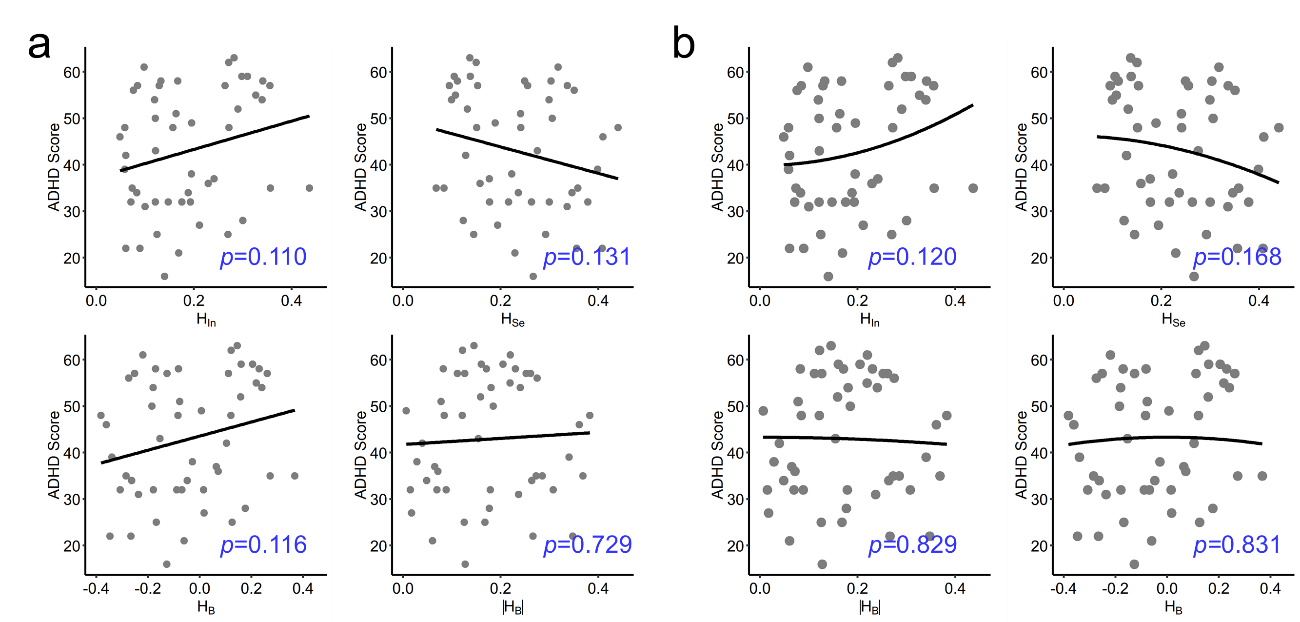


**Fig. S5 (a)** Linear and **(b)** quadratic relationships between ADHD score and brain measures.


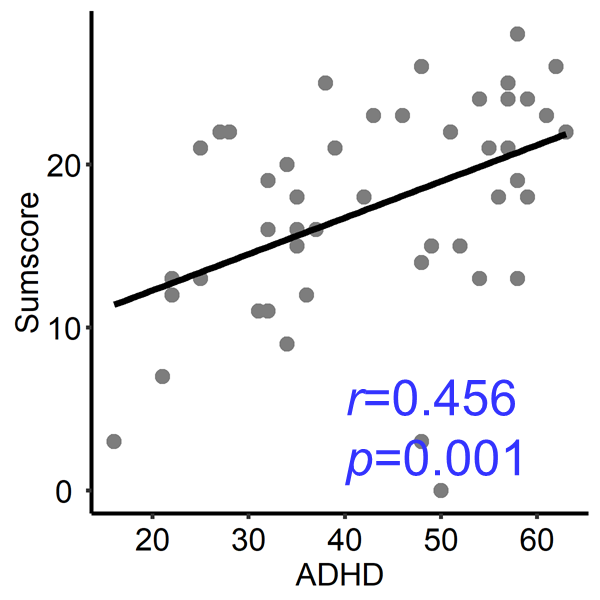


**Fig. S6** Correlation between ADHD and BD scores.
